# 100 most-cited publications in vascularized composite allotransplantation

**DOI:** 10.3389/frtra.2026.1745991

**Published:** 2026-02-18

**Authors:** Pharel Njessi, Carter J. Boyd, Palmina Petruzzo, Olivier Camuzard, Antoine Sicard, Rami Kantar, Eduardo D. Rodriguez, Elise Lupon

**Affiliations:** 1 Department of Plastic and Reconstructive Surgery, Institut Universitaire Locomoteur et du Sport, Pasteur 2 Hospital, University Côte d’Azur, Nice, France; 2 Laboratoire de PhysioMédecine Moléculaire, Université Côte D'Azur, Centre National de Recherche Scienctifique, Nice, France; 3 Hansjörg Wyss Department of Plastic Surgery, New York University Langone Health, New York, NY, United States; 4 Department of Transplantation, Hospital Edouard Herriot, Hospices Civils de Lyon, Lyon, France; 5 Department of Surgical Sciences, University of Cagliari, Cagliari, Italy; 6 Department of Nephrology, Dialysis and Kidney Transplantation, University Hospital of Nice, Nice, France

**Keywords:** bibliometric, citation analysis, face transplant, hand transplant, vascularized composite allograft (VCA), upper extremity transplantation

## Abstract

**Background:**

Citation analysis is a useful bibliometric tool to identify impactful publications and trace the evolution of a specialty or a technique. In the past three decades, the research on vascularized composite allotransplantation (VCA) has grown exponentially but very few studies have examined the most influential papers in this field.

**Methods:**

The Web of Science Core Collection database was searched for articles published from inception to August 4th, 2025. Titles, full authors' names, years of publication, source journals, regions of origin, and numbers of citations were recorded. VCA anatomical location, main topics, and citation density were determined. Articles were ranked based on number of citations and citation density; they were then categorized based on methodology, study design, and main topic.

**Results:**

The 100 most-cited articles on VCA were published between 1996 and 2018 with the number of citations per article ranging from 61 to 604 citations. There were 53 non-clinical studies, the most prevalent topics were outcomes and rehabilitation (*n* = 48 articles) and immunology (*n* = 37). Of the 75 studies evaluated using the Oxford Centre for Evidence-Based Medicine levels of evidence, most (*n* = 51) were classified as level 4.

**Discussion:**

This list of the top 100 most-cited articles highlights seminal and influential papers in VCA. It also demonstrates the relative novelty of this field with ongoing efforts in immunological research to allow its further expansion. The present study provides an understanding of VCA evolution while directing future clinical and preclinical studies.

## Introduction

Vascularized composite allotransplantation (VCA) has emerged over the past three decades as a reconstructive option for patients with devastating tissue loss not amenable to conventional autologous techniques. The first successful hand transplantation in 1998 marked a turning point for this field (1). Since then, VCA, including transplantation of hand, face, larynx, trachea, uterus, and abdominal wall has progressively demonstrated feasibility and functional benefits in carefully selected patients (2, 3). To date, fewer than 400 VCA procedures have been performed worldwide with the majority consisting of upper extremity (4) and face allotransplantations (5). The limited number of procedures performed underscores the rarity of this approach and the need for continued international collaboration.

The literature on VCA has grown substantially, covering basic immunology, surgical techniques, ethical debates, functional outcomes, and quality of life. However, the field remains heterogeneous, and the relatively low number of procedures worldwide makes it difficult for trainees, surgeons, and researchers to identify the studies that have shaped current practice. Citation analysis has been recognized as a valuable bibliometric tool to highlight impactful publications, trace the evolution of a technique, and provide insight into research priorities (6). Citation analyses have already been applied in related areas of transplantation and reconstructive surgery (7–11). To date, however, no bibliometric study has focused specifically on the most-cited publications in VCA. The aim of this bibliometric study was therefore to identify the 100 most-cited articles in VCA and to analyze their key characteristics.

## Materials and methods

### Search strategy

**Figure 1:**
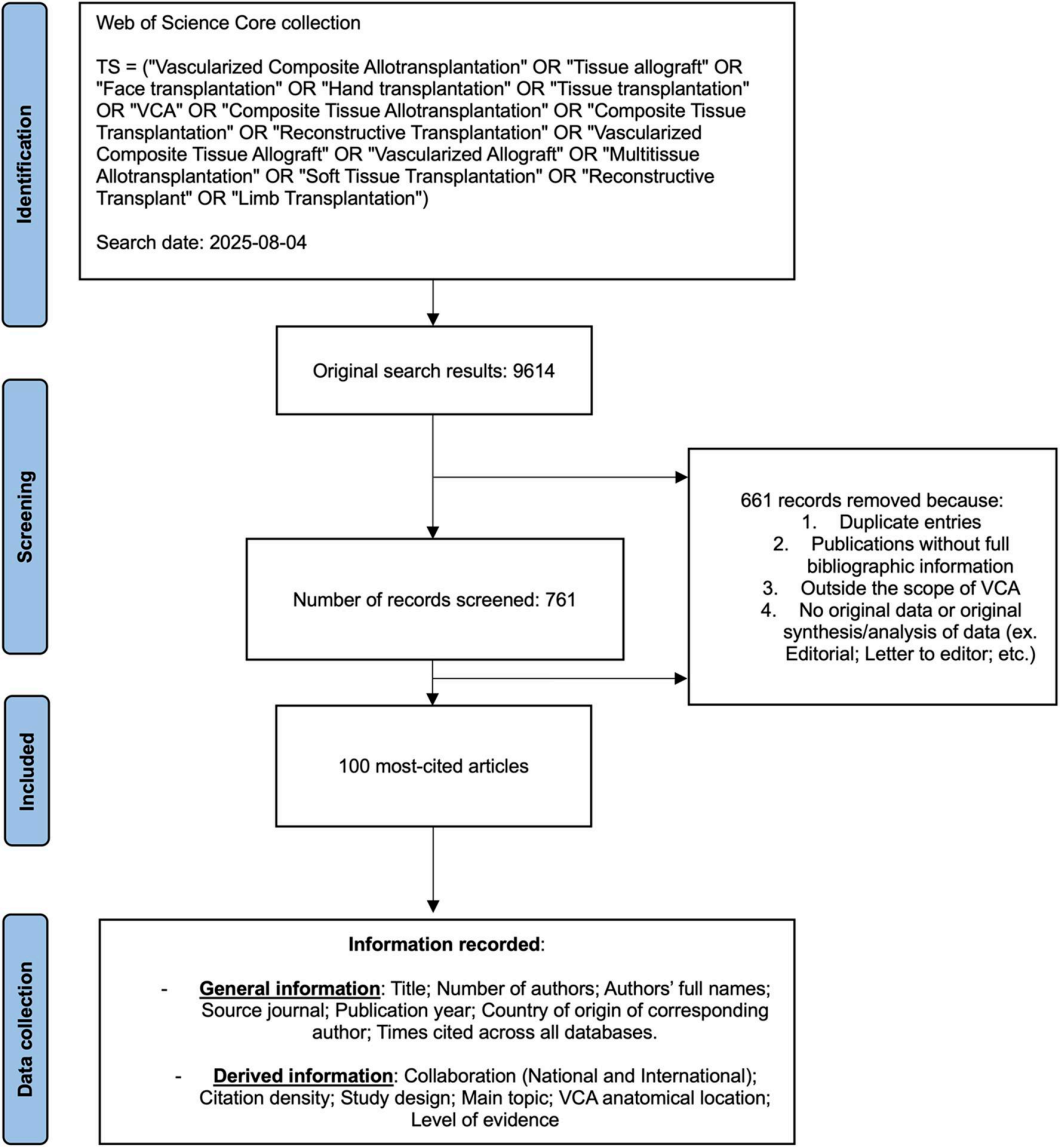
Flowchart depicting the creation of the database of the 100 most-cited VCA articles.

The 100 most-cited articles relating to VCA were extracted with no restrictions from all available journals through the online Web of Science Core Collection database on August 4th, 2025. The following key terms were used: “*Vascularized Composite Allotransplantation” OR “Tissue allograft” OR “Face transplantation” OR “Hand transplantation” OR “Tissue transplantation” OR “VCA” OR “Composite Tissue Allotransplantation” OR “Composite Tissue Transplantation” OR “Reconstructive Transplantation” OR “Vascularized Composite Tissue Allograft” OR “Vascularized Allograft” OR “Multi-tissue Allotransplantation” OR “Soft Tissue Transplantation” OR “Reconstructive Transplant” OR “Limb Transplantation”*. The initial search yielded 9614 records (Figure 1). The search results were then deduplicated and assessed for eligibility based on the following inclusion criteria: studies presenting original data, review articles synthesizing or analyzing original data. Exclusion criteria included: publications without full bibliographic information, studies outside the scope of VCA, articles with no original data or original synthesis or analysis of data (e.g., editorial, letter to editor, etc.) (Supplementary Material, S1). When different articles reported on the same patient case, such as the two initial reports on the first face transplantation (12, 13), they were included separately in the analysis. Among the Banff consensus papers, only the 2007 working classification of skin-containing composite tissue allograft pathology (14) was included as it is the only one specifically addressing VCA. If after full-text review, doubt remained about the eligibility of an article, the senior author (E.L.) was consulted for final decision. A total of 761 records were reviewed to reach the 100 most-cited VCA articles.

### Data collection and analysis

Data were collected and analyzed on a computerized spreadsheet (Microsoft Excel 2025, Microsoft Corporation, Wash.). For each of the included reports, the following data were recorded: title, full authors' names, year of publication, source journal, regions of origin and number of citations. The region of origin of the article refers to that of the corresponding author. The collaboration status and the VCA anatomical location were extracted from titles and abstracts and, when necessary, through full-text review. The citation density was calculated by the total number of citations divided by the years since the paper was published as previously described (15).

The articles were divided into non-clinical and clinical studies. Non-clinical articles included experimental studies on animal or on human cadavers or cells as well as ethical discussion, cost-utility analysis study, and review article. Clinical studies were categorized into randomized controlled trial, non-randomized controlled trial, cohort study, case series/report, and registry report. Finally, each study was classified into one of six topics: 1) Clinical and Surgical advances; 2) Immunology; 3) Preservation; 4) Technological innovation; 5) Outcomes and Rehabilitation; and 6) Ethical, Psychosocial, and Financial Aspects. Clinical articles and review studies were evaluated using the Oxford Center for Evidence-Based Medicine (OCEBM) (16) Levels of Evidence system (Supplementary Material, S2).

## Results

### Citation profiles and journal distribution

**Figure 2:**
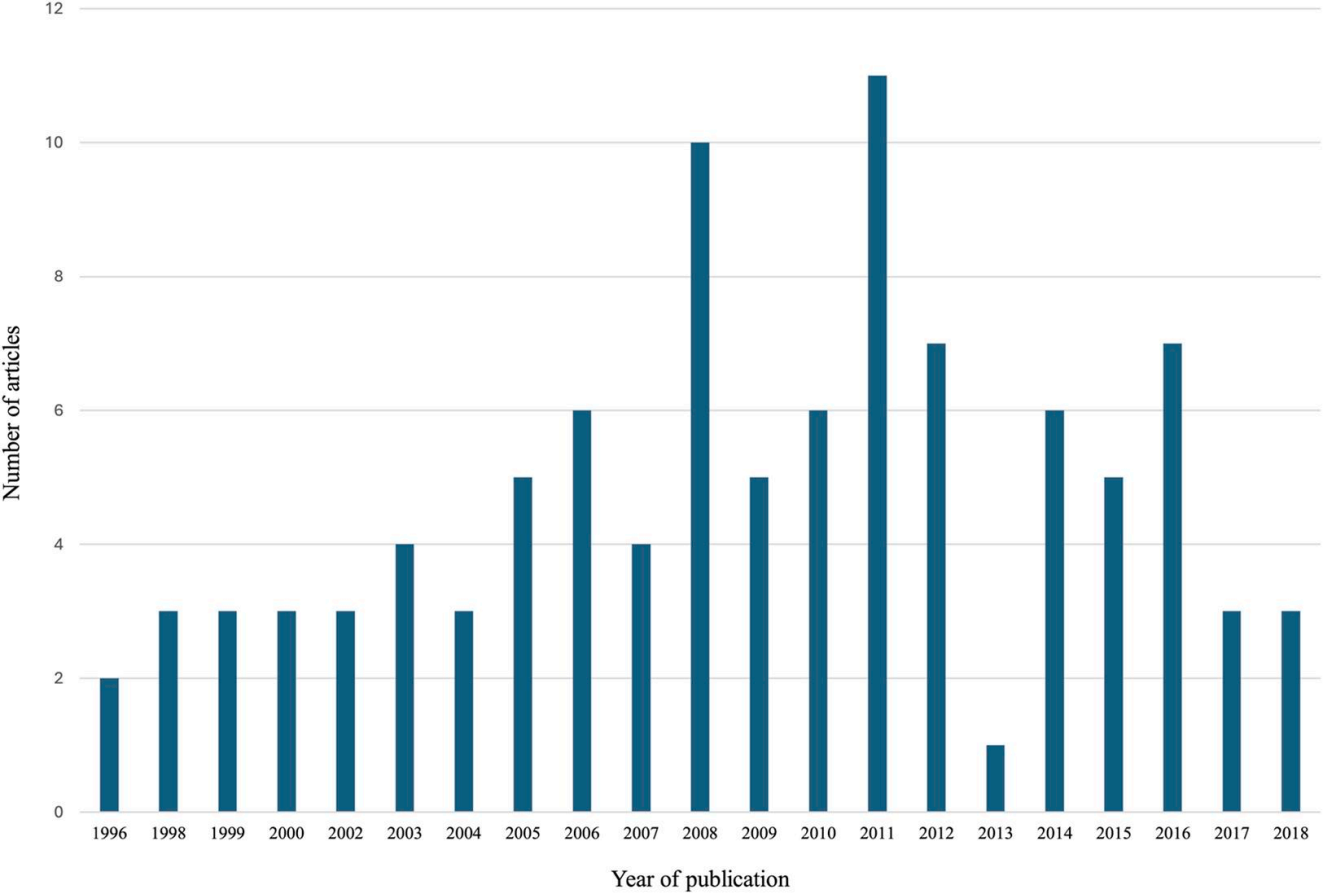
The number of articles published over the years among the 100 most-cited VCA articles.

**Figure 3:**
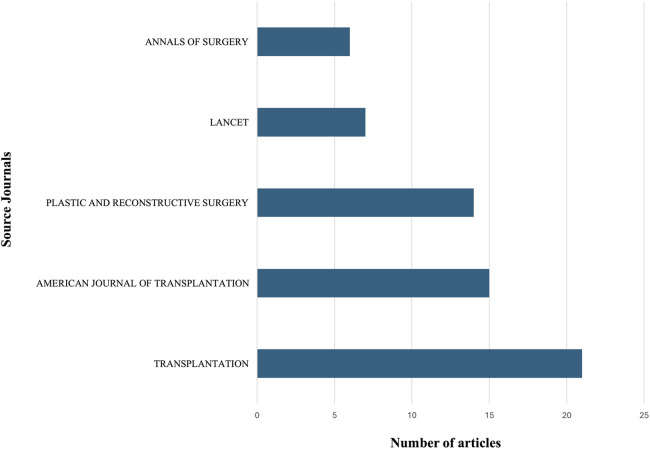
The five journals that contributed largest number of publications to the 100 most-cited VCA articles.

The 100 most-cited articles on VCA in descending order of citations are reported in Supplementary Material, S3. The combined number of citations received by the 100 most-cited VCA papers was 11,925. The number of citations per article ranged from 61 to 604 and the average citation density (CD) was 7.99 (range: 2.52–27.63). The most cited article, with 604 citations (CD, 23.23), was a case report on the first successful human hand transplant published by Dubernard et al. in 1999 (1). Devauchelle et al.'s case report on the first human face allograft, published in 2006 (12), was the second most cited article (525 citations) and had the highest citation density (CD, 27.63). The articles were published between 1996 (17, 18) and 2018 (19–21) with the highest number of articles (*n* = 11) occurring in 2011 (Figure 2). The oldest papers on the list were published two years before the first successful human VCA (1) by Buttemeyer et al. (18) and Benhaim et al. (17) and have accrued 148 and 79 citations, respectively. Both studies explored immunosuppressive treatments in rats' hindlimb models of VCA. Among the 29 journals that contributed to the top 100 most-cited VCA papers (Supplementary Material, S4), *Transplantation* published the highest number of articles (*n* = 21). Figure 3 shows the top five journals that contributed to the 100 most-cited VCA articles.

### Methodology, topic, and level of evidence

**Figure 4:**
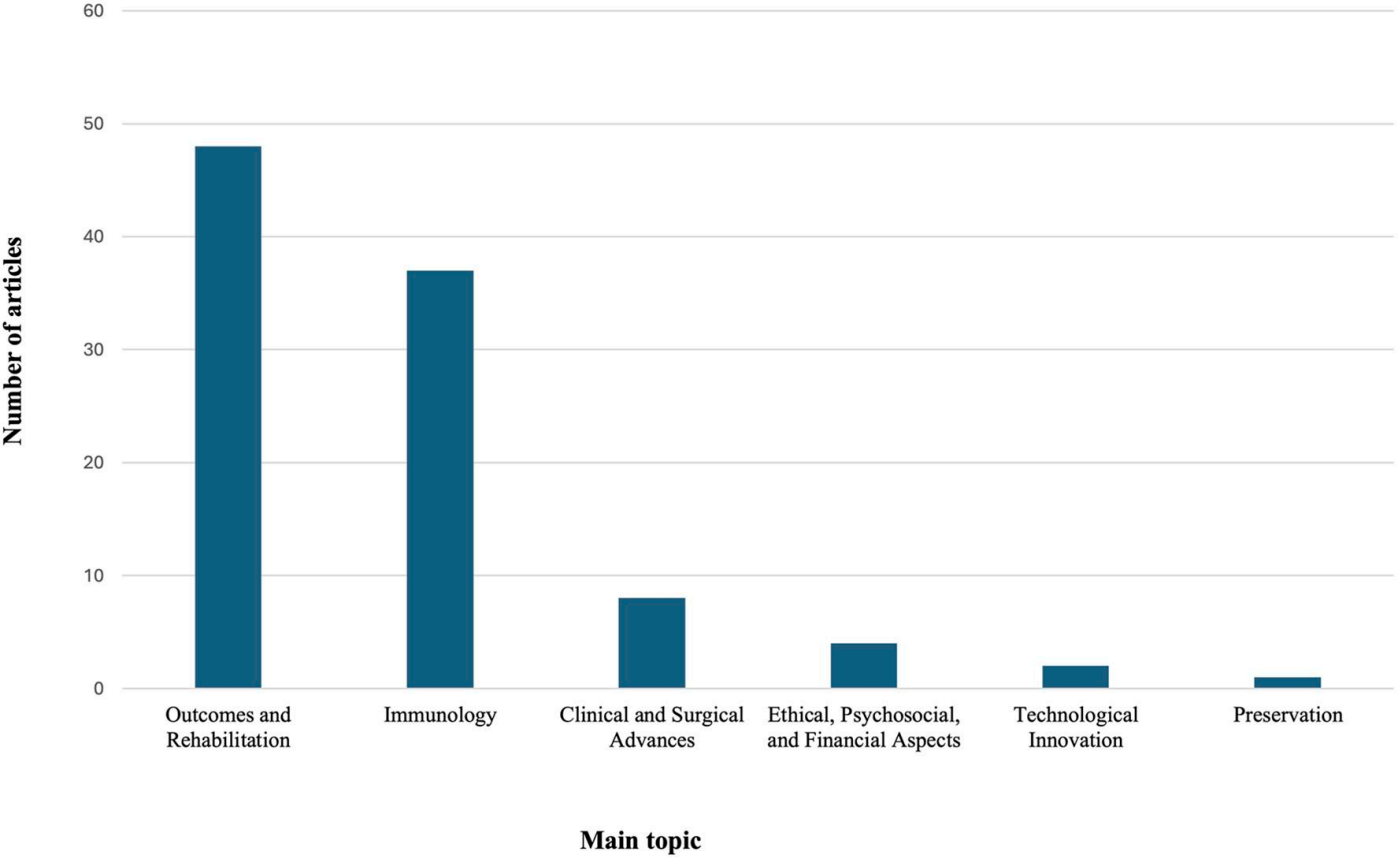
The topic of the top 100 most-cited VCA articles.

**Figure 5:**
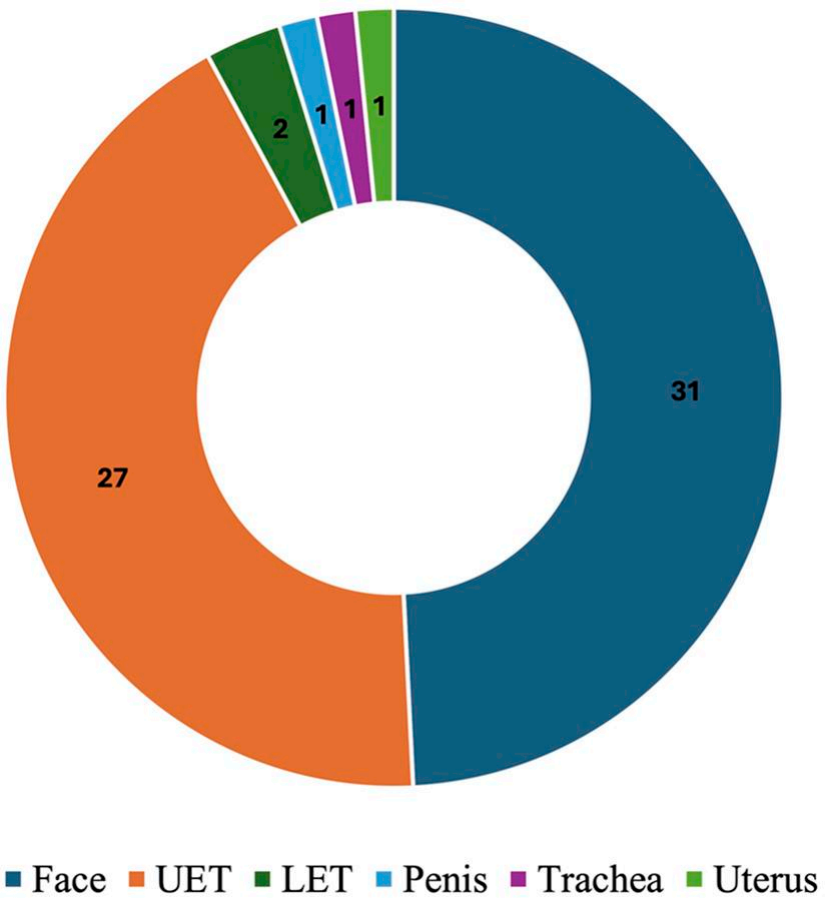
The VCA anatomic locations in the studies focusing on a single anatomic location among the 100 most-cited VCA articles. LET: Lower Extremity Transplantation; UET: Upper Extremity Transplantation.

Although the majority of the studies were non-clinical (*n* = 53), the most frequent study design was case report (*n* = 25). Experimental studies were common, especially preclinical animal studies (*n* = 22), with fewer studies involving human cadavers (*n* = 3) or human cells (*n* = 2) (Table 1). Most articles focused on outcomes and rehabilitation (*n* = 48). Immunology was the primary topic in 37 studies (Figure 4). Most articles focused one anatomical location (*n* = 63), particularly face (*n* = 31) and upper extremity allografts (*n* = 27) (Figure 5).

**Table 1:** The study design of the 100 most-cited VCA articles.

Rank	Study design	Frequency
1	Case report	25
2	Experimental (animal study)	22
3	Review	22
4	Case series	19
5	Experimental (cadaveric study)	3
6	Registry report	3
7	Experimental (human cell study)	2
8	Ethical discussion	2
9	Cost-utility analysis	1
10	Systematic Review	1

Of the 74 reports assessed using the OCEBM system, no study achieved level 1 or level 3 evidence. The most common level of evidence was level 4 (*n* = 51) followed by level 5 (*n* = 25) due to the high proportion of case reports/series and reviews. Only one paper, a systematic review on outcomes after hand VCA published by Landin et al. (22), achieved level 2 evidence (Table 2).

**Table 2:** The OCEBM level of evidence of the clinical, review, and cost-analysis studies among the 100 most-cited VCA articles.

Level of evidence	Study design
Level 1	0
Level 2	1
Level 3	0
Level 4	51
Level 5	25
Total	75

### Language, region of origin and collaborations

**Figure 6:**
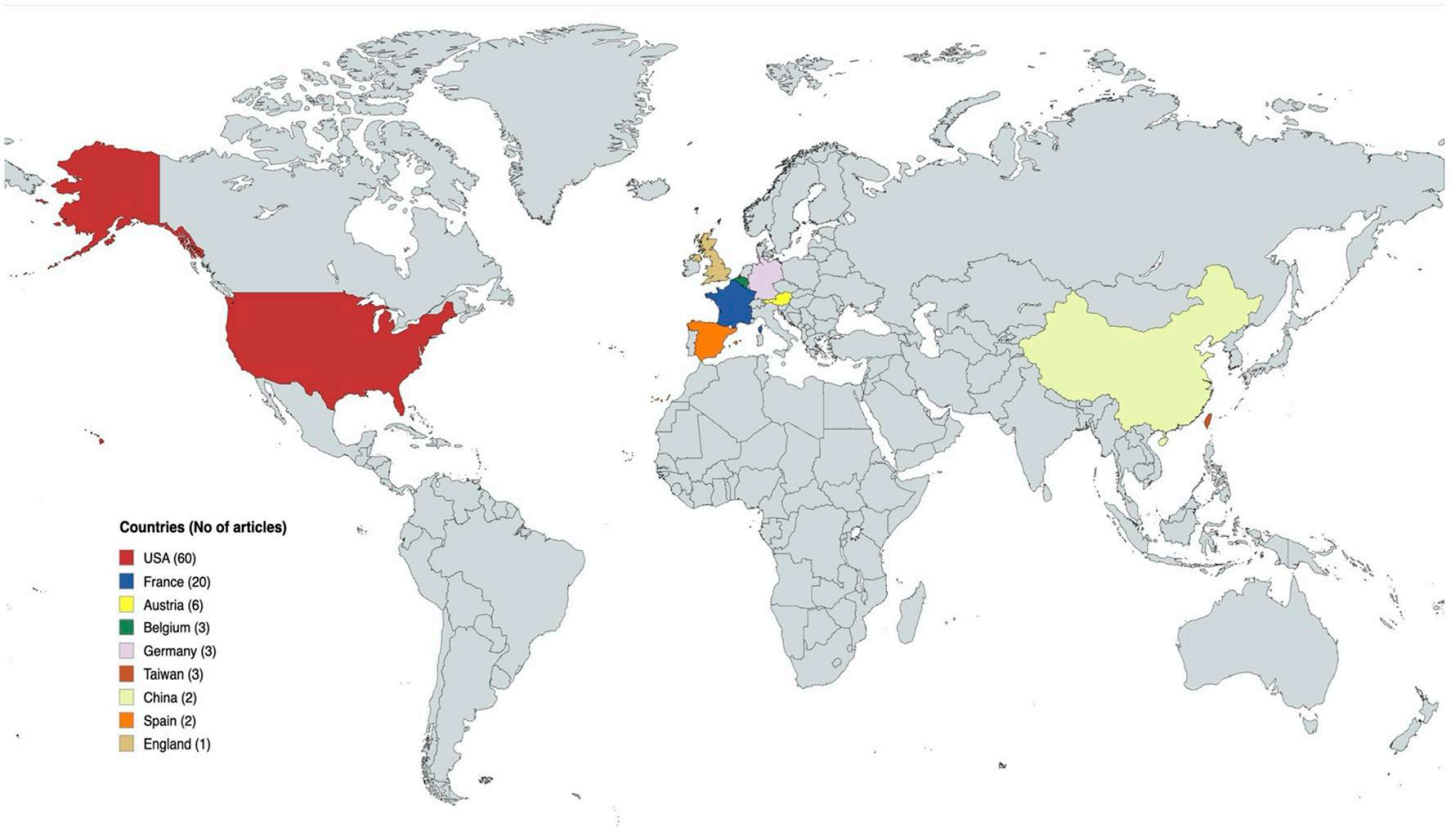
The geographic distribution of the origins of the 100 most-cited VCA articles.

All papers in the list were published in English. The region that contributed the highest number of publications was the United States of America (USA) (*n* = 60) followed by Europe (*n* = 35). Collaborations among institutions were common, with 53% of the top 100 articles involving collaborative efforts. International collaborations accounted for 35% of the included articles, whereas national collaborations represented 15%. Among international collaborations, the most common study designs were case reports (*n* = 11), review articles (*n* = 10), and case series (*n* = 7). Figure 6 presents the geographic distribution of the origins of the 100 most-cited VCA articles.

## Discussion

Among plastic surgery journals, *Plastic and Reconstructive Surgery* contributed the most articles, ranking third overall with 14 publications and following *Transplantation* (*n* = 21) and *The American Journal of Transplantation* (*n* = 15). This journal distribution demonstrates that VCAs are transplantations as well as reconstructive procedures, highlighting their multi-disciplinary nature. The French team led by Prof. Dubernard performed the first hand and face transplantations and consequently their articles are among the most cited (1, 2, 12). However, several teams from Europe and the USA have also greatly contributed to the development of the field by reporting their experience in VCAs. These findings are consistent with previous bibliometric studies reporting a high contribution from the United States across multiple specialties (15, 23, 24). Most articles explored upper extremity or face transplantations, which are the most common types of VCA (25) although uterus transplantation is rapidly expanding (26). Interestingly, while clinically UET dominates the VCA field, the face was the most represented anatomical location in the included studies. Although a citation bias favoring highly publicized procedures cannot be excluded, this finding could also reflect the landmark nature, ethical implications, and multidisciplinary impact of facial transplantation rather than its absolute procedural volume.

Still regarded as one of the major innovations within plastic surgery (27, 28), VCA is a relatively new field. Accordingly, most clinical publications consist of case reports and case series, with no comparative studies. This lack of comparative research explains the generally low level of evidence, with only one study (22) achieving level 2 according to the OCEBM system. Although a low level of evidence is common in plastic surgery literature (23, 29), VCA research is particularly vulnerable due to the limited number of procedures performed. Systematic data reporting and centralization through initiatives such as the *International Registry on Hand and Composite Tissue Transplantation* (30) could improve the quality of evidence in VCA outcomes. The relative novelty of this field is further reflected by the high number of preclinical studies among the 100 most-cited papers. Many VCA publications remained experimental, with a large proportion of preclinical animal studies. Most of these animal studies focus on immunology including tolerance induction strategies and immune treatment protocols. Collectively, they established the biological feasibility of VCA and provided the foundation for future clinical applications. The high prevalence of preclinical studies has also been observed in bibliometric analysis of face transplant literature (7). Likewise, the predominance of immunology-focused work among highly cited VCA publications parallels findings in solid organ transplant bibliometric studies, where immunological mechanisms, rejection, and immunosuppression are also recuring themes (31–33). This reflects the foundational role of immune responses in graft survival and transplant success.

Both the experimental nature of VCA procedures and the associated ethical considerations make controlled trials impossible; but this may change in the future. For example, the superiority of one rehabilitative procedure over the other is being investigated for arm/hand transplantation and prosthesis in upper limb reconstruction (34, 35). Moreover, although only one article on graft preservation (36) appeared among the top 100 VCA publications, interest in preservation strategies is likely to increase in the coming years. Extending preservation time could not only expand the VCA donor pool by enabling long-distance transport, it could also provide additional time for recipient conditioning in immune tolerance protocols (37). In brief, this list of the 100 most-cited articles indicates that clinical experience in VCA is still limited despite the first successful procedure dating back to almost three decades. Greater collaboration among teams will be essential to support further development of the field.

This study has several limitations. First, citation analysis is cross-sectional in nature, and although VCA procedures remain rare, research in this field continues to evolve rapidly. Thus, the list of the 100 most-cited VCA articles identified here is likely to change in the upcoming years as the field progresses. Second, the search was restricted to peer-reviewed research articles and did not include textbooks, lectures or non-peer reviewed papers. It is therefore difficult to truly appreciate the impact that other resources might have had on VCA literature. Third, even though citation count can be used as a surrogate marker of scientific impact, it is inherently subject to bibliometric biases. These include time-dependent citation advantage favoring older publications, self-citation practices, and the disproportionate influence of high-volume centers with sustained academic output (38, 39). In the present study, this limitation is reflected in our reliance on absolute citation counts and the observed highest publication year. Ranking based on the absolute citation may have disadvantaged papers with high citation density but relatively low total citation numbers. Likewise, the time-dependent citation advantage likely contributes to the observed peak around 2011, as articles from this period have had sufficient time to accumulate citations. These factors should be considered when interpreting citation-based rankings. Finally, it is important to acknowledge that this analysis was not designed to capture the full contribution of all surgical teams or countries performing VCAs. The contrast between the limited number of clinical procedures and the concentration of publications from a few centers underscores a persistent geographic imbalance. Countries such as India, China, or Turkey, where noteworthy clinical activity (2, 40) has occurred remain underrepresented in bibliometric studies. Scientific literature should accurately depict global VCA practice, including VCAs performed in all countries. Despite its limitations, this paper represents the first bibliometric analysis of the most-cited VCA articles. It helps identify influential papers and track the evolution of VCA.

In conclusion, this bibliometric study identified the 100 most-cited articles on VCA. Despite major advances, VCA procedures remain for the most part experimental, as reflected by the predominance of case reports (*n* = 25), case series (*n* = 19), and preclinical studies (*n* = 22) among the most cited articles. Improvements in immunology and graft preservation techniques could make more clinical studies possible leading to an expansion of this field. The articles included in this paper can help achieve this goal by informing learners and clinicians while guiding researchers.

## Supplementary material

The Supplementary Materialfor this article can be found online at: https://www.frontiersin.org/articles/10.3389/frtra.2026.1745991/full#supplementary-material
