# Supplementary material for: 100 most-cited publications in vascularized composite allotransplantation
Source: Front Transplant. 2026 Feb 18;5:1745991. doi: 10.3389/frtra.2026.1745991 (PMC12957187; doi:10.3389/frtra.2026.1745991)
Supplement: Supplementary file 1 [file Datasheet1.docx]

**Supplementary material**

**S1. Inclusion and exclusion criteria used for selection of the 100 most-cited VCA articles**

| **Inclusion criteria** | **Exclusion criteria** |
| --- | --- |
| 1. Articles primarily focused on VCA, including hand, face, abdominal wall, uterus, larynx, trachea, limb, or multicomponent soft tissue transplantation. 2. Publications addressing clinical experience, surgical technique, immunology, ethics, rehabilitation, or functional outcomes related to VCA. 3. Original articles (case reports, case series, cohort studies), review articles, ethical discussion and experimental research directly relevant to the field. 4. Articles written in English and indexed in the Web of Science database. | 1. Publications exclusively concerning **ovarian, testicular, corneal, neural, or isolated bone allotransplantation,** as these are generally considered outside the scope of VCA. 2. **Editorials, conference abstracts, and letters to the editor** 3. Duplicate entries and publications without full bibliographic information. |

**S2.** The Oxford Center for Evidence-Based Medicine (OCEBM) Levels of Evidence system*****


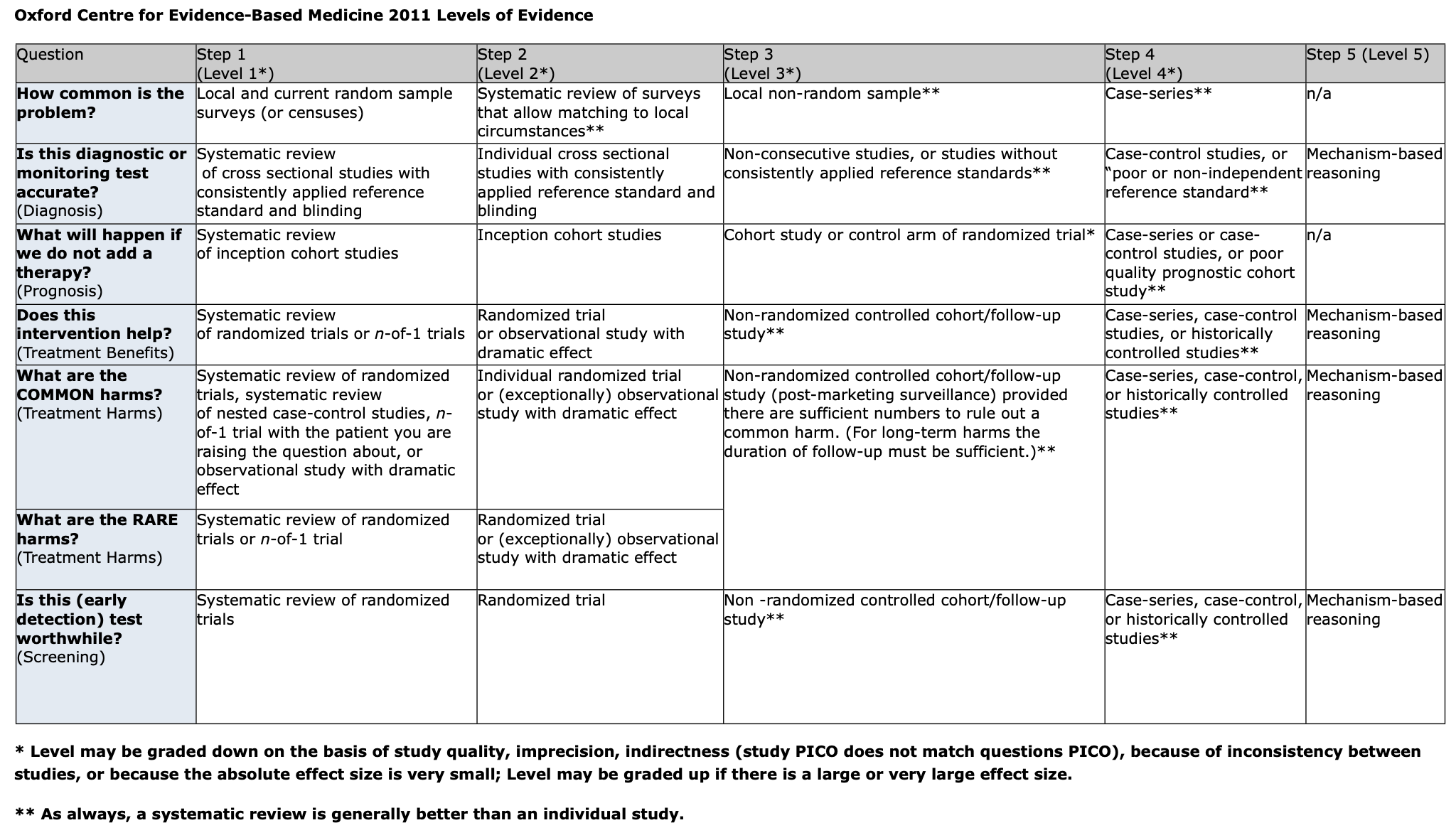


*Source: OCEBM Levels of Evidence Working Group. "The Oxford 2011 Levels of Evidence". Oxford Centre for Evidence-Based Medicine. <http://www.cebm.net/index.aspx?o=5653>. Accessed on: October 17, 2025.

**S3.** The characteristics of the 100 most-cited VCA articles

| **Rank** | **Title** | **Authors' full names** | **No of citations** | **Citation density** | **Study design** | **VCA anatomic location** | **Level of evidence** |
| --- | --- | --- | --- | --- | --- | --- | --- |
| 1 | Human hand allograft: report on first 6 months | Dubernard, JM; Owen, E; Herzberg, G; Lanzetta, M; Martin, X; Kapila, H; Dawahra, M; Hakim, NS | 604 | 23.23 | Case report | UET | Level 4 |
| 2 | First human face allograft: early report | Devauchelle, Bernard; Badet, Lionel; Lengele, Benoit; Morelon, Emmanuel; Testelin, Sylvie; Michallet, Mauricette; D'Hauthuille, Cedric; Dubernard, Jean-Michel | 525 | 27.63 | Case report | Face | Level 4 |
| 3 | The Banff 2007 working classification of skin-containing composite tissue Allograft Pathology | Cendales, L. C.; Kanitakis, J.; Schneeberger, S.; Burns, C.; Ruiz, P.; Landin, L.; Remmelink, M.; Hewitt, C. W.; Landgren, T.; Lyons, B.; Drachenberg, C. B.; Solez, K.; Kirk, A. D.; Kleiner, D. E.; Racusen, L. | 404 | 23.76 | Review | ≥ 2 | Level 5 |
| 4 | Successful hand transplantation - One-year follow-up | Jones, JW; Gruber, SA; Barker, JH; Breidenbach, WC | 353 | 14.12 | Case report | UET | Level 4 |
| 5 | Outcomes 18 months after the first human partial face transplantation | Dubernard, Jean-Michel; Lengele, Benoit; Morelon, Emmanuel; Testelin, Sylvie; Badet, Lionel; Moure, Christophe; Beziat, Jean-Luc; Dakpe, Stephanie; Kanitakis, Jean; D'Hauthuille, Cedric; El Jaafari, Assia; Petruzzo, Palmina; Lefrancois, Nicole; Taha, Farid; Sirigu, Angela; Di Marco, Giovanni; Carmi, Esther; Bachmann, Danielle; Cremades, Sophie; Giraux, Pascal; Burloux, Gabriel; Hequet, Olivier; Parquet, Nathalie; Frances, Camille; Michallet, Mauricette; Martin, Xavier; Devauchelle, Bernard | 337 | 18.72 | Case report | UET | Level 4 |
| 6 | Tracheal Allotransplantation after Withdrawal of Immunosuppressive Therapy | Delaere, Pierre; Vranckx, Jan; Verleden, Geert; De Leyn, Paul; Van Raemdonck, Dirk | 274 | 18.27 | Case report | Trachea | Level 4 |
| 7 | Near-total human face transplantation for a severely disfigured patient in the USA | Siemionow, Maria; Papay, Frank; Alam, Daniel; Bernard, Steven; Djohan, Risal; Gordon, Chad; Hendrickson, Mark; Lohman, Robert; Eghtesad, Bijan; Coffman, Kathy; Kodish, Eric; Paradis, Carmen; Avery, Robin; Fung, John | 266 | 16.63 | Case report | Face | Level 4 |
| 8 | Repair of the lower and middle parts of the face by composite tissue allotransplantation in a patient with massive plexiform neurofibroma: a 1-year follow-up study | Lantieri, Laurent; Meningaud, Jean-Paul; Grimbert, Philippe; Bellivier, Frank; Lefaucheur, Jean-Pascal; Ortonne, Nicolas; Benjoar, Marc-David; Lang, Philippe; Wolkenstein, Pierre | 257 | 15.12 | Case report | Face | Level 4 |
| 9 | Human facial allotransplantation: a 2-year follow-up study | Guo, Shuzhong; Han, Yon; Zhang, Xudong; Lu, Binglun; Yi, Chenggang; Zhang, Hui; Ma, Xianjie; Wang, Datai; Yang, Li; Fan, Xing; Liu, Yunjing; Lu, Kaihua; Li, Huiyuan | 236 | 13.88 | Case report | Face | Level 4 |
| 10 | Facial transplantation: the first 9 years | Khalifian, Saami; Brazio, Philip S.; Mohan, Raja; Shaffer, Cynthia; Brandacher, Gerald; Barth, Rolf N.; Rodriguez, Eduardo D. | 212 | 19.27 | Review | Face | Level 4 |
| 11 | Three Patients with Full Facial Transplantation | Pomahac, Bohdan; Pribaz, Julian; Eriksson, Elof; Bueno, Ericka M.; Diaz-Siso, J. Rodrigo; Rybicki, Frank J.; Annino, Donald J.; Orgill, Dennis; Caterson, Edward J.; Caterson, Stephanie A.; Carty, Matthew J.; Chun, Yoon S.; Sampson, Christian E.; Janis, Jeffrey E.; Alam, Daniel S.; Saavedra, Arturo; Molnar, Joseph A.; Edrich, Thomas; Marty, Francisco M.; Tullius, Stefan G. | 207 | 15.92 | Case series | Face | Level 4 |
| 12 | Hand and Upper Extremity Transplantation: An Update of Outcomes in the Worldwide Experience | Shores, Jaimie T.; Brandacher, Gerald; Lee, W. P. Andrew | 187 | 18.70 | Review | UET | Level 4 |
| 13 | Feasibility, Reproducibility, Risks and Benefits of Face Transplantation: A Prospective Study of Outcomes | Lantieri, L.; Hivelin, M.; Audard, V.; Benjoar, M. D.; Meningaud, J. P.; Bellivier, F.; Ortonne, N.; Lefaucheur, J.-P.; Gilton, A.; Suberbielle, C.; Marty, J.; Lang, P.; Grimbert, P. | 180 | 12.86 | Case series | Face | Level 4 |
| 14 | Upper-Extremity Transplantation Using a Cell-Based Protocol to Minimize Immunosuppression | Schneeberger, Stefan; Gorantla, Vijay S.; Brandacher, Gerald; Zeevi, Adriana; Demetris, Anthony J.; Lunz, John G.; Metes, Diana M.; Donnenberg, Albert D.; Shores, Jaimie T.; Dimartini, Andrea F.; Kiss, Joseph E.; Imbriglia, Joseph E.; Azari, Kodi; Goitz, Robert J.; Manders, Ernest K.; Nguyen, Vu T.; Cooney, Damon S.; Wachtman, Galen S.; Keith, Jonathan D.; Fletcher, Derek R.; Macedo, Camila; Planinsic, Raymond; Losee, Joseph E.; Shapiro, Ron; Starzl, Thomas E.; Lee, W. P. Andrew | 174 | 14.50 | Case series | UET | Level 4 |
| 15 | First live birth after uterus transplantation in the United States | Testa, G.; McKenna, G. J.; Gunby, R. T., Jr.; Anthony, T.; Koon, E. C.; Warren, A. M.; Putman, J. M.; Zhang, L.; dePrisco, G.; Mitchell, J. M.; Wallis, K.; Klintmalm, G. B.; Olausson, M.; Johannesson, L. | 173 | 24.71 | Case report | Uterus | Level 4 |
| 16 | Hand transplantation: Comparisons and observations of the first four clinical cases | Francois, CG; Breidenbach, WC; Maldonado, C; Kakoulidis, TP; Hodges, A; Dubernard, JM; Owen, E; Pei, GX; Ren, XP; Barker, JH | 157 | 6.28 | Case series | UET | Level 4 |
| 17 | First US Near-Total Human Face Transplantation: A Paradigm Shift for Massive Complex Injuries | Siemionow, Maria Z.; Papay, Frank; Djohan, Risal; Bernard, Steven; Gordon, Chad R.; Alam, Daniel; Hendrickson, Mark; Lohman, Robert; Eghtesad, Bijan; Fung, John | 149 | 9.93 | Case report | Face | Level 4 |
| 18 | Graft Vasculopathy in Clinical Hand Transplantation | Kaufman, C. L.; Ouseph, R.; Blair, B.; Kutz, J. E.; Tsai, T. M.; Scheker, L. R.; Tien, H. Y.; Moreno, R.; Ozyurekoglu, T.; Banegas, R.; Murphy, E.; Burns, C. B.; Zaring, R.; Cook, D. F.; Marvin, M. R. | 148 | 11.38 | Case series | UET | Level 4 |
| 19 | Rejection of the component tissues of limb allografts in rats immunosuppressed with FK-506 and cyclosporine | Buttemeyer, R; Jones, NF; Min, Z; Rao, U | 148 | 5.10 | Experimental (animal study) | N/A | N/A |
| 20 | Functional results of the first human double-hand transplantation | Dubernard, JM; Petruzzo, P; Lanzetta, M; Parmentier, H; Martin, X; Dawahra, M; Hakim, NS; Owen, E | 136 | 6.18 | Case report | UET | Level 4 |
| 21 | A single localized dose of enzyme-responsive hydrogel improves long-term survival of a vascularized composite allograft | Gajanayake, Thusitha; Olariu, Radu; Leclere, Franck M.; Dhayani, Ashish; Yang, Zijiang; Bongoni, Anjan K.; Banz, Yara; Constantinescu, Mihai A.; Karp, Jeffrey M.; Vemula, Praveen Kumar; Rieben, Robert; Voegelin, Esther | 132 | 12.00 | Experimental (animal study) | N/A | N/A |
| 22 | Full Face Transplant The First Case Report | Barret, Juan P.; Gavalda, Joan; Bueno, Javier; Nuvials, Xavier; Pont, Teresa; Masnou, Nuria; Colomina, Maria J.; Serracanta, Jordi; Arno, Anna; Huguet, Pere; Collado, Jose M.; Salamero, Pere; Moreno, Carlos; Deulofeu, Roser; Martinez-Ibanez, Vicenc | 131 | 9.36 | Case report | Face | Level 4 |
| 23 | Second report (1998-2006) of the International Registry of Hand and Composite Tissue Transplantation | Lanzetta, Marco; Petruzzo, Palmina; Dubernard, Jean Michel; Margreiter, Raimund; Schuind, Frederic; Breidenbach, Warren; Nolli, Roberta; Schneeberger, Stephan; van Holder, Carlo; Gorantla, Vijay S.; Pei, Guoxian; Zhao, Jinmin; Zhang, Xinying | 129 | 7.17 | Registry report | UET | Level 4 |
| 24 | Face transplant: long-term follow-up and results of a prospective open study | Lantieri, Laurent; Grimbert, Philippe; Ortonne, Nicolas; Suberbielle, Caroline; Bories, Dominique; Gil-Vernet, Salvador; Lemogne, Cedric; Bellivier, Frank; Lefaucheur, Jean Pascal; Schaffer, Nathaniel; Martin, Frederic; Meningaud, Jean Paul; Wolkenstein, Pierre; Hivelin, Mikael | 127 | 14.11 | Case series | Face | Level 4 |
| 25 | Restoration of Facial Form and Function After Severe Disfigurement from Burn Injury by a Composite Facial Allograft | Pomahac, B.; Pribaz, J.; Eriksson, E.; Annino, D.; Caterson, S.; Sampson, C.; Chun, Y.; Orgill, D.; Nowinski, D.; Tullius, S. G. | 127 | 9.07 | Case report | Face | Level 4 |
| 26 | The International Registry on Hand and Composite Tissue Transplantation | Lanzetta, M; Petruzzo, P; Margreiter, R; Dubernard, JM; Schuind, F; Breidenbach, W; Lucchina, S; Schneeberger, S; van Holder, C; Granger, D; Pei, GX; Zhao, JM; Zhang, X | 127 | 6.35 | Registry report | UET | Level 4 |
| 27 | The World's Experience With Facial Transplantation What Have We Learned Thus Far? | Gordon, Chad R.; Siemionow, Maria; Papay, Francis; Pryor, Landon; Gatherwright, James; Kodish, Eric; Paradis, Carmen; Coffman, Kathy; Mathes, David; Schneeberger, Stefan; Losee, Joseph; Serletti, Joseph M.; Hivelin, Mikael; Lantieri, Laurent; Zins, James E. | 122 | 7.63 | Review | Face | Level 4 |
| 28 | Split tolerance to a composite tissue allograft in a swine model | Mathes, DW; Randolph, MA; Solari, MG; Nazzal, JA; Nielsen, GP; Arn, JS; Sachs, DH; Lee, WPA | 119 | 5.41 | Experimental (animal study) | N/A | N/A |
| 29 | Generation and Transplantation of an Autologous Vascularized Bioartificial Human Tissue | Mertsching, Heike; Schanz, Johanna; Steger, Volker; Schandar, Markus; Schenk, Martin; Hansmann, Jan; Dally, Iris; Friedel, Godehard; Walles, Thorsten | 109 | 6.81 | Experimental (animal study) | N/A | N/A |
| 30 | Vascularized composite allotransplantation: current standards and novel approaches to prevent acute rejection and chronic allograft deterioration | Kueckelhaus, Maximilian; Fischer, Sebastian; Seyda, Midas; Bueno, Ericka M.; Aycart, Mario A.; Alhefzi, Muayyad; ElKhal, Abdallah; Pomahac, Bohdan; Tullius, Stefan G. | 108 | 12.00 | Review | ≥ 2 | Level 5 |
| 31 | Engineered composite tissue as a bioartificial limb graft | Jank, Bernhard J.; Xiong, Linjie; Moser, Philipp T.; Guyette, Jacques P.; Ren, Xi; Cetrulo, Curtis L.; Leonard, David A.; Fernandez, Leopoldo; Fagan, Shawn P.; Ott, Harald C. | 102 | 10.20 | Experimental (animal study) | N/A | N/A |
| 32 | The International Registry on Hand and Composite Tissue Transplantation | Petruzzo, Palmina; Lanzetta, Marco; Dubernard, Jean Michel; Margreiter, Raimund; Schuind, Frederic; Breidenbach, Warren; Nolli, Roberta; Schneeberger, Stephan; van Holder, Carlo; Kaufman, Christina; Jablecki, Jerzy; Landin, Luis; Cavadas, Pedro | 101 | 5.94 | Registry report | UET | Level 4 |
| 33 | Outcomes of the first 2 American hand transplants at 8 and 6 years posttransplant | Breidenbach, Warren C.; Gonzales, N. Ruben; Kaufman, Christina L.; Klapheke, Martin; Tobin, Gordon R.; Gorantla, Vijay S. | 101 | 5.94 | Case series | UET | Level 4 |
| 34 | On the ethics of facial transplantation research | Wiggins, OP; Barker, JH; Martinez, S; Vossen, M; Maldonado, C; Grossi, FV; Francois, CG; Cunningham, M; Perez-Abadia, G; Kon, M; Banis, JC | 101 | 4.81 | Ethical discussion | Face | Level 4 |
| 35 | First Human Face Transplantation: 5 Years Outcomes | Petruzzo, Palmina; Testelin, Sylvie; Kanitakis, Jean; Badet, Lionel; Lengele, Benoit; Girbon, Jean-Pierre; Parmentier, Helene; Malcus, Christophe; Morelon, Emmanuel; Devauchelle, Bernard; Dubernard, Jean-Michel | 100 | 7.69 | Case report | Face | Level 4 |
| 36 | Tolerance induction in composite facial allograft transplantation in the rat model | Demir, Y; Ozmen, S; Klimczak, A; Mukherjee, AL; Siemionow, M | 99 | 4.71 | Experimental (animal study) | N/A | N/A |
| 37 | Functional Outcomes of Face Transplantation | Fischer, S.; Kueckelhaus, M.; Pauzenberger, R.; Bueno, E. M.; Pomahac, B. | 98 | 9.80 | Case series | Face | Level 4 |
| 38 | Long-Term Follow-Up in Composite Tissue Allotransplantation: In-Depth Study of Five (Hand and Face) Recipients | Petruzzo, P.; Kanitakis, J.; Badet, L.; Pialat, J. -B.; Boutroy, S.; Charpulat, R.; Mouly, J.; Gazarian, A.; Lanzetta, M.; Brunet, M.; Devauchelle, B.; Testelin, S.; Martin, X.; Dubernard, J. M.; Morelon, E. | 97 | 6.93 | Case series | ≥ 2 | Level 4 |
| 39 | The Face Transplantation Update: 2016 | Sosin, Michael; Rodriguez, Eduardo D. | 95 | 10.56 | Review | Face | Level 5 |
| 40 | The Management of Antibody-Mediated Rejection in the First Presensitized Recipient of a Full-Face Allotransplant | Chandraker, A.; Arscott, R.; Murphy, G. F.; Lian, C. G.; Bueno, E. M.; Marty, F. M.; Rennke, H. G.; Milford, E.; Tullius, S. G.; Pomahac, B. | 94 | 8.55 | Case report | Face | Level 4 |
| 41 | Long-term survival of an extremity composite tissue allograft with FK506-mycophenolate mofetil therapy | Jones, JW; Üstüner, ET; Zdichavsky, M; Edelstein, J; Ren, XP; Maldonado, C; Ray, M; Jevans, AW; Breidenbach, WC; Gruber, SA; Barker, JH | 94 | 3.62 | Experimental (animal study) | N/A | N/A |
| 42 | Clinicopathologic monitoring of the skin and oral mucosa of the first human face allograft:: Report on the first eight months | Kanitakis, Jean; Badet, Lionel; Petruzzo, Palmina; Beziat, Jean Luc; Morelon, Emmanuel; Lefrancois, Nicole; Frances, Camille; Claudy, Alain; Martin, Xavier; Lengele, Benoit; Testelin, Sylvie; Devauchelle, Bernard; Dubernard, Jean Michel | 92 | 4.84 | Case report | Face | Level 4 |
| 43 | Face Transplantation: Partial Graft Loss of the First Case 10 Years Later | Morelon, E.; Petruzzo, P.; Kanitakis, J.; Dakpe, S.; Thaunat, O.; Dubois, V.; Choukroun, G.; Testelin, S.; Dubernard, J. -M.; Badet, L.; Devauchelle, B. | 91 | 11.38 | Case report | Face | Level 4 |
| 44 | Composite Tissue Vasculopathy and Degeneration Following Multiple Episodes of Acute Rejection in Reconstructive Transplantation | Unadkat, J. V.; Schneeberger, S.; Horibe, E. H.; Goldbach, C.; Solari, M. G.; Washington, K. M.; Gorantla, V. S.; Cooper, G. M.; Thomson, A. W.; Lee, W. P. Andrew | 91 | 6.07 | Experimental (animal study) | N/A | N/A |
| 45 | A cadaver study in preparation for facial allograft transplantation in humans: Part I. What are alternative sources for total facial defect coverage? | Siemionow, M; Unal, S; Agaoglu, G; Sari, A | 90 | 4.74 | Experimental (cadaveric study) | Face | Level 5 |
| 46 | Mesenchymal Stem Cells Prolong Composite Tissue Allotransplant Survival in a Swine Model | Kuo, Yur-Ren; Goto, Shigeru; Shih, Hsiang-Shun; Wang, Feng-Sheng; Lin, Chien-Chih; Wang, Chun-Ting; Huang, Eng-Yen; Chen, Chao-Long; Wei, Fu-Chan; Zheng, Xin Xiao; Lee, W. P. Andrew | 90 | 5.63 | Experimental (animal study) | N/A | N/A |
| 47 | Outcomes after hand and upper extremity transplantation | Shores, Jaimie T.; Malek, Veronika; Lee, W. P. Andrew; Brandacher, Gerald | 89 | 11.13 | Review | UET | Level 5 |
| 48 | Face Transplantation: Outcomes, Concerns, Controversies, and Future Directions | Siemionow, Maria; Ozturk, Can | 89 | 6.85 | Review | Face | Level 5 |
| 49 | Face transplantation: A review of the technical, immunological, psychological and clinical issues with recommendations for good practice | Morris, Peter; Bradley, Andrew; Doyal, Len; Earley, Michael; Hagen, Patricia; Milling, Martin; Rumsey, Nichola | 89 | 4.94 | Review | Face | Level 5 |
| 50 | A double-hand transplant can be worth the effort! | Margreiter, R; Brandacher, G; Ninkovic, M; Steurer, W; Kreczy, A; Schneeberger, S | 88 | 3.83 | Case report | UET | Level 4 |
| 51 | Vascular Considerations in Composite Midfacial Allotransplantation | Pomahac, Bohdan; Lengele, Benoit; Ridgway, Emily B.; Matros, Evan; Andrews, Brian T.; Cooper, Jason S.; Kutz, Richard; Pribaz, Julian J. | 87 | 5.80 | Experimental (cadaveric study) | Face | Level 5 |
| 52 | Acute rejection in vascularized composite allotransplantation | Fischer, Sebastian; Lian, Christine G.; Kueckelhaus, Maximilian; Strom, Terry B.; Edelman, Elazer R.; Clark, Rachel A.; Murphy, George F.; Chandraker, Anil K.; Riella, Leonardo V.; Tullius, Stefan G.; Pomahac, Bohdan | 87 | 7.91 | Review | ≥ 2 | Level 5 |
| 53 | Long-term composite tissue allograft survival in a porcine model with cyclosporine/mycophenolate mofetil therapy | Üstüner, ET; Zdichavsky, M; Ren, X; Edelstein, J; Maldonado, C; Ray, M; Jevans, AW; Breidenbach, WC; Gruber, SA; Barker, JH; Jones, JW | 87 | 3.22 | Experimental (animal study) | N/A | N/A |
| 54 | Concerns about human hand transplantation in the 21st century | Jones, NF | 86 | 3.74 | Ethical discussion | UET | Level 5 |
| 55 | Allogeneic vascularized transplantation of human femoral diaphyses and total knee joints -: First clinical experiences | Hofmann, GO; Kirschner, MH; Wagner, FD; Brauns, L; Gonschorek, O; Bühren, V | 86 | 3.19 | Case series | LET | Level 4 |
| 56 | An Update on Facial Transplantation Cases Performed between 2005 and 2010 | Siemionow, Maria; Ozturk, Can | 84 | 6.00 | Review | Face | Level 5 |
| 57 | First forearm transplantation: Outcome at 3 years | Schneeberger, S.; Ninkovic, M.; Gabl, M.; Ninkovic, M.; Hussl, H.; Rieger, M.; Loescher, W.; Zelger, B.; Brandacher, G.; Bonatti, H.; Hautz, T.; Boesmueller, C.; Piza-Katzer, H.; Margreiter, R. | 84 | 4.67 | Case report | UET | Level 4 |
| 58 | Mesenchymal Stem Cells Enhance Nerve Regeneration in a Rat Sciatic Nerve Repair and Hindlimb Transplant Model | Cooney, Damon S.; Wimmers, Eric G.; Ibrahim, Zuhaib; Grahammer, Johanna; Christensen, Joani M.; Brat, Gabriel A.; Wu, Lehao W.; Sarhane, Karim A.; Lopez, Joseph; Wallner, Christoph; Furtmueller, Georg J.; Yuan, Nance; Pang, John; Sarkar, Kakali; Lee, W. P. Andrew; Brandacher, Gerald | 84 | 9.33 | Experimental (animal study) | N/A | N/A |
| 59 | Total Face, Eyelids, Ears, Scalp, and Skeletal Subunit Transplant: A Reconstructive Solution for the Full Face and Total Scalp Burn | Sosin, Michael; Ceradini, Daniel J.; Levine, Jamie P.; Hazen, Alexes; Staffenberg, David A.; Saadeh, Pierre B.; Flores, Roberto L.; Sweeney, Nicole G.; Bernstein, G. Leslie; Rodriguez, Eduardo D. | 83 | 9.22 | Case report | Face | Level 4 |
| 60 | Self-renewal capacity of human epidermal Langerhans cells: observations made on a composite tissue allograft | Kanitakis, Jean; Morelon, Emmanuel; Petruzzo, Palmina; Badet, Lionel; Dubernard, Jean-Michel | 83 | 5.93 | Experimental (human study) | N/A | N/A |
| 61 | Composite tissue allotransplantation of the hand and face: a new frontier in transplant and reconstructive surgery | Gander, Brian; Brown, Charles S.; Vasilic, Dalibor; Furr, Allen; Banis, Joseph C., Jr.; Cunningham, Michael; Wiggins, Osborne; Maldonado, Claudio; Whitaker, Iain; Perez-Abadia, Gustavo; Frank, Johannes M.; Barker, John H. | 83 | 4.37 | Review | ≥ 2 | Level 5 |
| 62 | Science of composite tissue allotransplantation | Swearingen, Bruce; Ravindra, Kadiyala; Xu, Hong; Wu, Shengli; Breidenbach, Warren C.; Ildstad, Suzanne T. | 82 | 4.82 | Review | ≥ 2 | Level 5 |
| 63 | Clinical experience in allogeneic vascularized bone and joint allografting | Hofmann, GO; Kirschner, MH | 81 | 3.24 | Case series | LET | Level 4 |
| 64 | Clinicopathological Findings of Chronic Rejection in a Face Grafted Patient | Petruzzo, Palmina; Kanitakis, Jean; Testelin, Sylvie; Pialat, Jean-Baptiste; Buron, Fanny; Badet, Lionel; Thaunat, Olivier; Devauchelle, Bernard; Morelon, Emmanuel | 80 | 8.00 | Case report | Face | Level 4 |
| 65 | Biomarker evaluation of face transplant rejection: association of donor T cells with target cell injury | Lian, Christine Guo; Bueno, Ericka M.; Granter, Scott R.; Laga, Alvaro C.; Saavedra, Arturo P.; Lin, William M.; Susa, Joseph S.; Zhan, Qian; Chandraker, Anil K.; Tullius, Stefan G.; Pomahac, Bohdan; Murphy, George F. | 80 | 7.27 | Experimental (human study) | N/A | N/A |
| 66 | Achievements and Challenges in Facial Transplantation | Rifkin, William J.; David, Joshua A.; Plana, Natalie M.; Kantar, Rami S.; Diaz-Siso, J. Rodrigo; Gelb, Bruce E.; Ceradini, Daniel J.; Rodriguez, Eduardo D. | 79 | 11.29 | Review | Face | Level 5 |
| 67 | Vascularized Composite Allograft Tolerance Across MHC Barriers in a Large Animal Model | Leonard, D. A.; Kurtz, J. M.; Mallard, C.; Albritton, A.; Duran-Struuck, R.; Farkash, E. A.; Crepeau, R.; Matar, A.; Horner, B. M.; Randolph, M. A.; Sachs, D. H.; Huang, C. A.; Cetrulo, C. L., Jr. | 79 | 7.18 | Experimental (animal study) | N/A | N/A |
| 68 | Modulation of Immune Response and T-Cell Regulation by Donor Adipose-Derived Stem Cells in a Rodent Hind-Limb Allotransplant Model | Kuo, Yur-Ren; Chen, Chien-Chang; Goto, Shigeru; Lee, I-Te; Huang, Chong-Wei; Tsai, Chia-Chun; Wang, Chun-Ting; Chen, Chao-Long | 79 | 5.64 | Experimental (animal study) | N/A | N/A |
| 69 | Use of combination of low-dose cyclosporine and RS-61443 in a rat hindlimb model of composite tissue allotransplantation | Benhaim, P; Anthony, JP; Ferreira, L; Borsanyi, JP; Mathes, SJ | 79 | 2.72 | Experimental (animal study) | N/A | N/A |
| 70 | Achievements and challenges in composite tissue allotransplantation | Schneeberger, Stefan; Landin, Luis; Jableki, Jerzy; Butler, Peter; Hoehnke, Christoph; Brandacher, Gerald; Morelon, Emmanuel | 78 | 5.57 | Review | ≥ 2 | Level 5 |
| 71 | Chronic Rejection in Human Vascularized Composite Allotransplantation (Hand and Face Recipients): An Update | Kanitakis, J; Petruzzo, P; Badet, L; Gazarian, A; Thaunat, O; Testelin, S; Devauchelle, B; Dubernard, JM; Morelon, E | 77 | 8.56 | Review | ≥ 2 | Level 5 |
| 72 | Induction of tolerance in composite-tissue allografts | Siemionow, M; Ortak, T; Izycki, D; Oke, R; Cunningham, B; Prajapati, R; Zins, JE | 77 | 3.35 | Experimental (animal study) | N/A | N/A |
| 73 | Atypical acute rejection after hand transplantation | Schneeberger, S.; Gorantla, V. S.; van Riet, R. P.; Lanzetta, M.; Vereecken, P.; van Holder, C.; Rorive, S.; Remmelink, M.; Le Moine, A.; Abramowicz, D.; Zelgeri, B.; Kaufman, C. L.; Breidenbach, W. C.; Margreiter, R.; Schuind, F. | 76 | 4.47 | Case series | UET | Level 4 |
| 74 | Hand transplantation in the United States: Experience with 3 patients | Ravindra, Kadiyala V.; Buell, Joseph F.; Kaufman, Christina L.; Blair, Brenda; Marvin, Michael; Nagubandi, Ravi; Breidenbach, Warren C. | 76 | 4.47 | Case series | UET | Level 4 |
| 75 | Face Transplant Graft Procurement: A Preclinical and Clinical Study | Meningaud, Jean-Paul; Paraskevas, Antoine; Ingallina, Fabio; Bouhana, Eric; Lantieri, Laurent | 75 | 4.41 | Case report | Face | Level 4 |
| 76 | Molecular Markers and Targeted Therapy of Skin Rejection in Composite Tissue Allotransplantation | Hautz, T.; Zelger, B.; Grahammer, J.; Krapf, C.; Amberger, A.; Brandacher, G.; Landin, L.; Mueller, H.; Schoen, M. P.; Cavadas, P.; Lee, A. W. P.; Pratschke, J.; Margreiter, R.; Schneeberger, S. | 74 | 4.93 | Experimental (animal study) | N/A | N/A |
| 77 | Cytomegalovirus-related complications in human hand transplantation | Schneeberger, S; Lucchina, S; Lanzetta, M; Brandacher, G; Bösmüller, C; Steurer, W; Baldanti, F; Dezza, C; Margreiter, R; Bonatti, H | 74 | 3.70 | Case series | UET | Level 4 |
| 78 | Composite tissue allotransplantation and reconstructive surgery - First clinical applications | Petit, F; Minns, AB; Dubernard, JM; Hettiaratchy, S; Lee, WPA | 73 | 3.32 | Review | ≥ 2 | Level 5 |
| 79 | Hand transplantation: Pertinent data and future outlook | Lee, WPA; Mathes, DW | 72 | 2.77 | Review | UET | Level 5 |
| 80 | Functional tolerance following face transplantation in the rat | Siemionow, M; Gozel-Ulusal, B; Ulusal, AE; Ozmen, S; Izyckj, D; Zins, JE | 68 | 3.09 | Experimental (animal study) | N/A | N/A |
| 81 | Donor-specific tolerance induction in composite tissue allografts | Foster, RD; Fan, L; Niepp, M; Kaufman, C; McCalmont, T; Ascher, N; Ildstad, S; Anthony, JP | 68 | 2.52 | Experimental (animal study) | N/A | N/A |
| 82 | Composite tissue allotransplantation - a new era in plastic surgery? | Hettiaratchy, S; Randolph, MA; Petit, F; Lee, WPA; Butler, PEM | 68 | 3.24 | Review | ≥ 2 | Level 5 |
| 83 | World Experience After More Than a Decade of Clinical Hand Transplantation: Update on the Innsbruck Program | Hautz, Theresa; Engelhardt, Timm O.; Weissenbacher, Annemarie; Kumnig, Martin; Zelger, Bettina; Rieger, Michael; Rumpold, Gerhard; Pierer, Gerhard; Ninkovic, Marina; Gabl, Markus; Piza-Katzer, Hildegunde; Pratschke, Johann; Margreiter, Raimund; Brandacher, Gerald; Schneeberger, Stefan | 67 | 4.79 | Case series | ≥ 2 | Level 4 |
| 84 | An Economic Analysis of Hand Transplantation in the United States | Chung, Kevin C.; Oda, Takashi; Saddawi-Konefka, Daniel; Shauver, Melissa J. | 67 | 4.47 | Cost-utility analysis | UET | N/A |
| 85 | Composite tissue allotransplantation: Development of a preclinical model in nonhuman primates | Cendales, LC; Xu, H; Bacher, J; Eckhaus, MA; Kleiner, DE; Kirk, AD | 67 | 3.35 | Experimental (animal study) | N/A | N/A |
| 86 | Prolongation of Composite Tissue Allotransplant Survival by Treatment with Bone Marrow Mesenchymal Stem Cells Is Correlated with T-Cell Regulation in a Swine Hind-Limb Model | Kuo, Yur-Ren; Chen, Chien-Chang; Shih, Hsiang-Shun; Goto, Shigeru; Huang, Chong-Wei; Wang, Chun-Ting; Chen, Chao-Long; Wei, Fu-Chan | 67 | 4.79 | Experimental (animal study) | N/A | N/A |
| 87 | Status 5 years after bilateral hand transplantation | Schneeberger, S; Ninkovic, M; Piza-Katzer, H; Gabl, M; Hussl, H; Rieger, M; Loescher, W; Zelger, B; Brandacher, G; Ninkovic, M; Bonatti, H; Boesmueller, C; Mark, W; Margreiter, R | 66 | 3.47 | Case report | UET | Level 4 |
| 88 | Outcomes with respect to disabilities of the upper limb after hand allograft transplantation: a systematic review | Landin, Luis; Bonastre, Jorge; Casado-Sanchez, Cesar; Diez, Jesus; Ninkovic, Marina; Lanzetta, Marco; del Bene, Massimo; Schneeberger, Stefan; Hautz, Theresa; Lovic, Aleksandar; Leyva, Francisco; Garcia-de-Lorenzo, Abelardo; Casado-Perez, Cesar | 65 | 5.00 | Systematic Review | UET | Level 2 |
| 89 | The Effect of Ex Situ Perfusion in a Swine Limb Vascularized Composite Tissue Allograft on Survival up to 24 Hours | Ozer, Kagan; Rojas-Pena, Alvaro; Mendias, Christopher L.; Bryner, Benjamin S.; Toomasian, Cory; Bartlett, Robert H. | 65 | 7.22 | Experimental (animal study) | N/A | N/A |
| 90 | The challenge of dermatopathological diagnosis of composite tissue allograft rejection: a review | Kanitakis, Jean | 64 | 3.76 | Review | ≥ 2 | Level 5 |
| 91 | Minimizing immunosuppression in hand transplantation | Brandacher, Gerald; Lee, W. P. Andrew; Schneeberger, Stefan | 63 | 4.85 | Review | UET | Level 5 |
| 92 | Pathological score for the evaluation of allograft rejection in human hand (composite tissue) allotransplantation | Kanitakis, J; Petruzzo, P; Jullien, D; Badet, L; Dezza, MC; Claudy, A; Lanzetta, M; Hakim, N; Owen, E; Dubernard, JM | 63 | 3.15 | Case series | UET | Level 4 |
| 93 | Penis Transplantation First US Experience | Cetrulo, Curtis L., Jr.; Li, Kai; Salinas, Harry M.; Treiser, Matthew D.; Schol, Ilse; Barrisford, Glen W.; McGovern, Francis J.; Feldman, Adam S.; Grant, Michael T.; Tanrikut, Cigdem; Lee, Jeffrey H.; Ehrlichman, Richard J.; Holzer, Paul W.; Choy, Garry M.; Liu, Raymond W.; Ng, Zhi Yang; Lellouch, Alexandre G.; Kurtz, Josef M.; Austen, William G., Jr.; Winograd, Jonathan M.; Bojovic, Branko; Eberlin, Kyle R.; Rosales, Ivy A.; Colvin, Robert B.; Ko, Dicken S. C. | 63 | 9.00 | Case report | Penis | Level 4 |
| 94 | The decade of face transplant outcomes | Siemionow, Maria | 62 | 7.75 | Review | Face | Level 5 |
| 95 | A Report of 15 Hand Allotransplantations in 12 Patients and Their Outcomes in China | Pei, Guoxian; Xiang, Dayong; Gu, Liqiang; Wang, Gang; Zhu, Lijun; Yu, Lixin; Wang, Huijun; Zhang, Xinying; Zhao, Jinmin; Jiang, Cunzhi; Wang, Zengtao; Liu, Wei | 62 | 4.77 | Case series | UET | Level 4 |
| 96 | An Anatomical Study of External Carotid Artery Vascular Territories in Face and Midface Flaps for Transplantation | Banks, Nia D.; Hui-Chou, Helen G.; Tripathi, Satyen; Collins, Brendan J.; Stanwix, Matthew G.; Nam, Arthur J.; Rodriguez, Eduardo D. | 62 | 3.88 | Experimental (cadaveric study) | Face | N/A |
| 97 | World Experience After More Than a Decade of Clinical Hand Transplantation: Update from the Louisville Hand Transplant Program | Kaufman, Christina L.; Breidenbach, Warren | 61 | 4.36 | Case series | UET | Level 4 |
| 98 | Complex facial reconstruction by vascularized composite allotransplantation: The first Belgian case | Roche, Nathalie A.; Vermeersch, Hubert F.; Stillaert, Filip B.; Peters, Kevin T.; De Cubber, Jan; Van Lierde, Kristiane; Rogiers, Xavier; Colenbie, Luc; Peeters, Patrick C.; Lemmens, Gilbert M. D.; Blondeel, Phillip N. | 61 | 6.10 | Case report | Face | Level 4 |
| 99 | Development and maintenance of donor-specific chimerism in semi-allogenic and fully major histocompatibility complex mismatched facial allograft transplants | Siemionow, M; Demir, Y; Mukherjee, A; Klimczak, A | 61 | 3.05 | Experimental (animal study) | N/A | N/A |
| 100 | Composite tissue allotransplantation: Classification of clinical acute skin rejection | Cendales, LC; Kirk, AD; Moresi, JM; Ruiz, P; Kleiner, DL | 61 | 3.21 | Case series | ≥ 2 | Level 4 |

**S4. The** 29 journals that contributed to the top 100 most-cited VCA papers

| **Rank** | **Journals** | **Frequency** |
| --- | --- | --- |
| 1 | TRANSPLANTATION | 21 |
| 2 | AMERICAN JOURNAL OF TRANSPLANTATION | 15 |
| 3 | PLASTIC AND RECONSTRUCTIVE SURGERY | 14 |
| 4 | LANCET | 7 |
| 5 | ANNALS OF SURGERY | 6 |
| 6 | JOURNAL OF HAND SURGERY-AMERICAN VOLUME | 4 |
| 7 | NEW ENGLAND JOURNAL OF MEDICINE | 4 |
| 8 | TRANSPLANT INTERNATIONAL | 4 |
| 9 | CURRENT OPINION IN ORGAN TRANSPLANTATION | 2 |
| 10 | HAND CLINICS | 2 |
| 11 | JOURNAL OF MATERIALS SCIENCE-MATERIALS IN MEDICINE | 2 |
| 12 | SURGERY | 2 |
| 13 | AMERICAN JOURNAL OF BIOETHICS | 1 |
| 14 | AMERICAN JOURNAL OF SURGERY | 1 |
| 15 | ANNALS OF PLASTIC SURGERY | 1 |
| 16 | BIOMATERIALS | 1 |
| 17 | BRITISH JOURNAL OF PLASTIC SURGERY | 1 |
| 18 | EUROPEAN JOURNAL OF DERMATOLOGY | 1 |
| 19 | EXPERIMENTAL DERMATOLOGY | 1 |
| 20 | EXPERT REVIEW OF CLINICAL IMMUNOLOGY | 1 |
| 21 | JOURNAL OF CRANIOFACIAL SURGERY | 1 |
| 22 | JOURNAL OF CUTANEOUS PATHOLOGY | 1 |
| 23 | JOURNAL OF PLASTIC RECONSTRUCTIVE AND AESTHETIC SURGERY | 1 |
| 24 | MICROSURGERY | 1 |
| 25 | MODERN PATHOLOGY | 1 |
| 26 | SCIENCE TRANSLATIONAL MEDICINE | 1 |
| 27 | SCIENTIFIC REPORTS | 1 |
| 28 | TRANSPLANT IMMUNOLOGY | 1 |
| 29 | TRANSPLANTATION PROCEEDINGS | 1 |
